# The Effectiveness of COVID-19 Vaccines in Improving the Outcomes of Hospitalized COVID-19 Patients

**DOI:** 10.7759/cureus.21485

**Published:** 2022-01-22

**Authors:** Welaia A Alsaffar, Albatool A Alwesaibi, Mousa J Alhaddad, Zainab K Alsenan, Hawra J Alsheef, Saleh H Alramadan, Hassan A Aljassas, Mohammed A Alsaghirat, Hassan J Alzahrani

**Affiliations:** 1 Internal Medicine, Dammam Medical Complex, Dammam, SAU; 2 Gastroenterology, Dammam Medical Complex, Dammam, SAU

**Keywords:** intubation, icu, admission, vaccine, infection, covid-19

## Abstract

Background

With the rapid spread of coronavirus disease 2019 (COVID-19), most countries took extreme measures to control the disease. Equitable access to safe and effective vaccines is critical to ending the COVID-19 pandemic. The Ministry of Health program in Saudi Arabia aimed to cover 17.4 million adults (70% of the adult population of Saudi Arabia) by the third quarter of 2021. We investigated the impact of the COVID-19 vaccine on the clinical course and outcomes of the admitted confirmed COVID-19 patients, in comparison to non-vaccinated patients.

Methodology

A retrospective cross-sectional record review was conducted for all hospitalized confirmed COVID-19 patients at Dammam Medical Complex (Eastern Province, Saudi Arabia) from June to July 2021. Two groups were studied according to the vaccination status (i.e., vaccinated and non-vaccinated). Information regarding comorbidities, length of stay, high oxygen requirements, ICU admission, and mortality data were collected and analyzed using the Python programming language (version 3.7.6) with the use of SciPy library (1.4.1) and Statsmodels module (v0.11.1).

Results

The sample included a total of 260 admitted confirmed COVID-19 cases. The mortality was significantly lower in the vaccinated group versus the non-vaccinated group with an odds ratio (OR) of 0.378 (CI 0.154-0.928). In addition, the OR of ICU admission was 0.476 (CI 0.218-1.042) and OR of endotracheal intubation was 0.561 (CI 0.249-1.265), but these did not reach statistical significance. We also detected a statistically significant relationship between mortality - regardless of vaccination status - and age ≥ 65 years (P=0.000, OR=7.51; 95%CI 3.13 to 18.04), chronic kidney disease (P=0.010, OR=5.62; CI 1.52 to 20.79), and renal transplant (P=0.037, OR=10.17; CI 1.15 to 89.76).

Of note, most of our vaccinated patients received only a single dose (85%).

Conclusion

There is a significant reduction in mortality cases as well as less complicated hospital courses among the vaccinated group, in spite of the fact that most of our admitted patients had only a single vaccine shot.

Suboptimal response to the vaccines was observed in the elder, chronic kidney disease, and renal transplant patients, hence the poorer outcomes in comparison to other patients.

## Introduction

Understanding of the coronavirus disease 2019 (COVID-19) is evolving; the extent of the spread and the severity of illness prompted widespread attempts to identify and recognize the nature of the disease. Rapid identification and sequencing of the virus allowed scientists - with the help of research centers and biotechnical companies - to begin developing preventive vaccines quickly. Vaccination is now considered the most promising approach for ending or containing the coronavirus disease 2019 (COVID-19) pandemic [1]. COVID19 vaccine development had progressed through preclinical evaluation and underwent three clinical phase trials. Pfizer-BioNTech and Oxford-AstraZeneca COVID-19 vaccines have shown high efficacy against disease in phase 3 clinical trials and are now being used in national vaccination programs in the UK and several other countries [2,3]. In the United States, Pfizer-BioNTech, Moderna, and Janssen COVID-19 vaccines have been granted emergency use authorization (EUA) for the prevention of COVID-19 [4,5]. They are all effective at preventing and reducing the risk of different variants of the COVID-19 virus, especially the severe form of the disease [6,7]. They are also valuable and proven to be safe in the elderly, frail, immunosuppressed, and vulnerable nursing home populations [8-10]. The Pfizer/BioNTech provides immunogenicity for at least 119 days after the first vaccination [11]. A two-dose regimen of Pfizer/BioNTech vaccine had 95% efficacy (95% CI 90.3-97.6) in preventing symptomatic COVID-19 at or after day 7 following the second dose in placebo-controlled multinational trials [12]. On August 23, 2021, the US Food and Drug Administration approved the first COVID-19 vaccine, which is Pfizer/BioNTech. The mass roll-out of the first doses of the Pfizer/ BioNTech and Oxford-AstraZeneca vaccines was associated with substantial reductions in the risk of hospital admission due to COVID-19 in Scotland. In the prospective cohort study, the first dose of the Pfizer/ BioNTech vaccine was associated with a vaccine effect of 91% (95% CI 85-94) for reduced COVID-19 hospital admission at 28-34 days post-vaccination. Vaccine effect at the same time interval for the Oxford -AstraZeneca vaccine was 88% (95% CI 75-94) [13]. Saudi authorities established a system responsible for continually monitoring national and international updates, raising awareness, and taking proper actions to contain the spread of this disease.

In January of 2021, the Kingdom of Saudi Arabia has distributed the vaccines to all parts of the kingdom with eased access to achieve equity, as well as decelerate the spread of COVID-19 and improve its clinical outcomes. There are three vaccines approved for use against SARS-CoV-2: Oxford-AstraZeneca, Pfizer/BioNTech, and Moderna [14,15]. Until July 25, 2021, there have been 515,949 confirmed cases of COVID-19 with 8,155 deaths, reported to WHO. As of July 30, 2021, 53.7% of the population received at least one dose of the COVID-19 vaccine and 22.7% were fully vaccinated [16].

Our hospital - Dammam Medical Complex - is considered the main and front-line COVID-19 center in the Eastern Province of Saudi Arabia, since the beginning of the pandemic, where most of the COVID-19 patients in the region were admitted. Therefore, we are conducting a retrospective observational study showing the positive impact and effectiveness of COVID-19 vaccines at decreasing mortality, ICU admission, and the need for high oxygen supplementation, in comparison to non-vaccinated hospitalized patients.

## Materials and methods

Dammam Medical Complex (IRB) has approved the protocol and issued approval IM-01 after careful review. Approval is given for one year from the date of this letter (July 9, 2021). This is to confirm that DMC IRB operated in accordance with NCBE regulations and ICH GCP E6 R2.

Patient selection

We conducted a retrospective record review of all consecutive hospitalized patients in Dammam Medical Complex (Eastern Province, Saudi Arabia), who had tested positive for COVID-19 by polymerase chain reaction (PCR).

Inclusion criteria

All confirmed COVID-19 patients aged 18 and older were admitted to Dammam Medical Complex from June to July 2021.

Data collection

We used the patient’s medical record numbers (MRNs) to collect the data from the electronic hospital system: patient’s age, gender, presence or absence of chronic diseases (e.g., diabetes mellitus, hypertension, heart disease, lung disease, BMI, and cancer), oxygen requirement, intubation, ICU admission, hospital length of stay, and disposition. The study was approved by the Institutional Review Board of Dammam Medical Complex.

Patient’s vaccination status

We divided the patients into two groups according to their vaccination status. Individuals who received at least one dose of COVID-19 vaccine (BNT162b2 or Oxford-AstraZeneca) were placed in the “vaccinated group”; individuals who did not receive any vaccine dose were placed in the “non-vaccinated group.” In addition, the date of vaccination, number of doses, and the date of acquiring the infection were obtained.

Statistical analysis

The data were analyzed using the Python programming language (version 3.7.6) with the use of the SciPy library (1.4.1) and Statsmodels module (v0.11.1). Descriptive statistics (i.e., mean, standard deviation, count, and percentage) were calculated as necessary. Categorical variables were compared with the Chi-square test and continuous variables were compared with the two-sample t-test. Multivariate logistic regression models were created with death, intubation, ICU admission, and the need for high supplementary oxygen (10 L or more) or ventilation support as dependent variables. The models were adjusted for the male sex, age of 65 years or above, vaccination status, and major comorbid conditions (including diabetes mellitus, hypertension, bronchial asthma, chronic kidney disease, active cancer, and history of kidney transplantation). The odds ratios and confidence intervals were reported. A p-value of less than 0.05 was assumed to indicate statistical significance. All data were used only for research purposes.

## Results

The clinical characteristics of the patients are presented in Table 1. The records from a total of 260 patients with confirmed COVID-19 infection were analyzed, with a mean age of 51.72 ±15.1 years. Only 37.69% (n=98) received a COVID-19 vaccine; of them, only 5.77% (n=15) had two shots of the vaccine. Roughly half (58.85%) of the sample were males. The most common comorbidities were diabetes mellitus (40.0%, n=104), hypertension (36.15%, n=94), and morbid obesity (27.31%, n=71). Other chronic conditions include heart disease, chronic kidney disease, and obstructive lung diseases (11.15%, 7.69%, and 7.31%, respectively). Our study also included a small number of immunocompromised patients who had been diagnosed with an oncological disease (2.69%, n=7), and those who underwent solid organ transplantation (2.69%, n=7).

**Table 1:** Patients’ demographics (n = 260)

Characteristic	Vaccinated, n=98 (%)	Non-vaccinated. n=62 (%)
Gender		
F	32 (32.65%)	87 (53.7%)
M	66 (67.35%)	75 (46.3%)
Age ≥ 65 years	18 (18.37%)	33 (20.37%)
Diabetes Mellitus	48 (48.98%)	56 (34.57%)
Hypertension	40 (40.82%)	54 (33.33%)
Bronchial Asthma or Chronic Obstructive Pulmonary Disease	11 (11.22%)	8 (4.94%)
Chronic Kidney Disease	9 (9.18%)	11 (6.79%)
Heart Diseases	13 (13.26%)	16 (9.87)
Ischemic Heart	7 (7.14%)	9 (5.55%)
Heart Failure	2 (2.04%)	6 (3.7%)
Others	4 (4.08%)	1 (0.61%)
Body Mass Index (BMI) ≥ 30	25 (25.51%)	46 (28.4%)
Sickle Cell Disease	1 (1.02%)	2 (1.23%)
Active Cancer	2 (2.04%)	5 (3.08%)
Colon	1 (1.02%)	2 (1.23%)
Leukemia	1 (1.02%)	0 (0.00%)
Lymphoma	0 (0.00%)	1 (0.61%)
Breast	0 (0.00%)	1 (0.61%)
Renal	0 (0.00%)	1 (0.61%)
Post Organ Transplant	6 (2.69%)	1 (0.61%)
Renal	5 (5.1%)	1 (0.61%)
Liver	1 (1.02%)	0 (0.00%)
Vaccinated		
Yes	98 (37.69%)	
No	162 (62.31%)	
COVID-19 Vaccination Doses		
0	162 (62.31%)	
1	83 (31.92%)	
2	15 (5.77%)	

A comparison between vaccinated vs. non-vaccinated patients is shown in Table 2. We found that the vaccinated group had a better hospital course and outcomes; with lower rates of ICU admission, intubation, and death (15.31% vs. 22.84%, 14.29% vs.19.75%, and 13.27% vs. 21.6%, respectively). Significant P-values were found in the renal transplant group (P=0.02), diabetic (P=0.03), and surprisingly male patients (P=0.04).

**Table 2:** Comparison between vaccinated and none-vaccinated patients * Requiring non-invasive mechanical ventilation or 10 L or more of supplemental oxygen.

Characteristic	Non-Vaccinated (n=162)	Vaccinated (n=98)	P-value
Male Gender, count (%)	87 (53.7%)	66 (67.35%)	0.04
Age, mean ± SD	51.12±15.48	52.71±14.47	0.41
Age ≥ 65 years, count (%)	33 (20.37%)	18 (18.37%)	0.81
Diabetes Mellitus, count (%)	56 (34.57%)	48 (48.98%)	0.03
Hypertension, count (%)	54 (33.33%)	40 (40.82%)	0.28
Bronchial Asthma or Chronic Obstructive Pulmonary Disease , count (%)	8 (4.94%)	11 (11.22%)	0.10
Chronic Kidney Disease, count (%)	11 (6.79%)	9 (9.18%)	0.64
Heart Diseases, count (%)	16 (9.88%)	13 (13.27%)	0.52
Body Mass Index (BMI) ≥ 30, count (%)	46 (28.4%)	25 (25.51%)	0.72
Sickle Cell Disease, count (%)	2 (1.23%)	1 (1.02%)	0.66
Post Organ Transplant, count (%)	1 (0.62%)	6 (6.12%)	0.02
High Oxygen Need*, count (%)	69 (42.59%)	35 (35.71%)	0.33
ICU Admission, count (%)	37 (22.84%)	15 (15.31%)	0.19
Intubation, count (%)	32 (19.75%)	14 (14.29%)	0.34
Death, count (%)	35 (21.6%)	13 (13.27%)	0.13
Admission To Death, mean ± SD	12.49±8.13	16.23±7.27	0.15
Admission To Discharge, mean ± SD	8.87±9.62	9.78±8.95	0.49

To assess the outcomes among each patient’s characteristics listed in Table 3, multivariate logistic regression analysis was conducted. Results showed the following significant findings: age ≥ 65 years was associated with higher mortality rates (P=0.000, odds ratio =7.510; 95%CI 3.127-18.04), intubation (P=0.002, odds ratio=3.744; 95%CI 1.631-8.593), and ICU admission (P=0.002, odds ratio=3.539; 95%CI 1.601-7.823). Chronic Kidney disease were also noted to be directly correlated with mortality (P=0.010, odds ratio=5.616; 95%CI 1.517-20.792), intubation (P=0.099, odds ratio=2.822; 95%CI 0.822-9.694), and ICU admission (P=0.229, odds ratio=2.085; 95%CI 0.629-6.913). Renal transplant population was also at a higher mortality risk (P=0.037, odds ratio=10.166; 95%CI 1.151-89.755), intubation (P=0.048, odds ratio=8.295; 95%CI 1.016-67.751), and ICU care (P=0.039, odds ratio=8.406; 95%CI 1.119-63.143). Vaccination showed a significant correlation with survival, where even one dose of the vaccine was found to be protective (P=0.034, odds ratio=0.378; 95%CI 0.154-0.928).

**Table 3:** Multivariate Logistic Regression of Mortality and Other Indicators of Severity

Mortality				Intubation			ICU Admission			High Oxygen Needs		
Characteristic	P-value	Odds ratio	95% CI	P-value	Odds ratio	95% CI	P-value	Odds ratio	95% CI	P-value	Odds ratio	95% CI
Male Gender	0.037	0.438	0.201-0.952	0.069	0.508	0.245-1.055	0.148	0.601	0.301-1.199	0.797	1.074	0.622-1.854
Age 65 ≥ years	0.000	7.510	3.127-18.04	0.002	3.744	1.631-8.593	0.002	3.539	1.601-7.823	0.032	2.175	1.071-4.416
Diabetes Mellitus	0.078	2.065	0.921-4.63	0.446	1.351	0.623-2.929	0.639	1.194	0.569-2.505	0.296	1.363	0.763-2.437
Hypertension	0.075	0.408	0.152-1.093	0.479	0.726	0.299-1.762	0.736	0.867	0.378-1.987	0.226	0.666	0.345-1.286
Bronchial Asthma or COPD	0.060	3.402	0.948-12.207	0.145	2.385	0.741-7.677	0.056	2.972	0.973-9.075	0.191	1.947	0.717-5.289
Chronic Kidney Disease	0.01	5.616	1.517-20.792	0.099	2.822	0.822-9.694	0.229	2.085	0.629-6.913	0.13	2.367	0.775-7.227
Heart Diseases	0.811	1.142	0.384-3.393	0.762	0.846	0.287-2.497	0.739	1.185	0.436-3.222	0.786	0.884	0.364-2.149
BMI ≥ 30	0.260	1.592	0.709-3.576	0.138	1.772	0.832-3.775	0.26	1.521	0.733-3.158	0.415	1.283	0.705-2.336
Sickle Cell Disease	0.739	1.722	0.071-42.002	0.629	2.012	0.118-34.291	0.763	1.56	0.087-28.008	0.844	0.778	0.064-9.52
Active Cancer	0.950	1.071	0.125-9.216	0.484	0.402	0.031-5.161	0.413	0.342	0.026-4.455	0.857	0.861	0.169-4.383
Renal Transplant	0.037	10.166	1.151-89.755	0.048	8.295	1.016-67.751	0.039	8.406	1.119-63.143	0.112	6.417	0.646-63.729
Vaccination	0.034	0.378	0.154-0.928	0.164	0.561	0.249-1.265	0.063	0.476	0.218-1.042	0.127	0.644	0.366-1.133

Due to our small sample size, we demonstrated no statistical significance regarding mortality in the vaccinated and non-vaccinated patients in the subgroup analysis shown in Table 4.

**Table 4:** Mortality differences between vaccinated and non-vaccinated patients

Patient group	Vaccinated patients			Non-vaccinated patients			P-value
	Total	Died	Percentage	Total	Died	Percentage	
Elderly (>= 65 years)	18	6	33.33%	33	18	54.55%	0.2473
Diabetes Mellitus	48	8	16.67%	56	20	35.71%	0.0498
Hypertension	40	6	15.00%	54	18	33.33%	0.0757
Bronchial Asthma or Chronic Obstructive Pulmonary Diseases	11	2	18.18%	8	5	62.50%	0.1348
Chronic Kidney Diseases	9	4	44.44%	11	7	63.64%	0.6843
Heart Diseases	13	2	15.38%	16	7	43.75%	0.2155
Renal Transplant	5	3	60.00%	1	1	100.00%	0.6985
Body Mass Index (BMI) ≥ 30	25	6	24.00%	46	14	30.43%	0.7645

Table 5 compares the effect of one vs. two vaccine shots. The percentage of survival after one dose was 88% (73 out of 83 patients), and 80% (12 out of 15 patients) after the second shot.

**Table 5:** Patient’s outcomes after single vs. two vaccine doses

Vaccine Doses	Total patients	Died	Survived
1	83	10 (12%)	73 (88%)
2	15	3 (20%)	12 (80%)

## Discussion

In our retrospective cross-sectional study, we evaluated the impact of COVID-19 vaccination and its effectiveness in the inpatient setting, in terms of decreasing mortality, the need for ICU admission, intubation, and high oxygen demand related to severe COVID-19 infection. We investigated 260 hospitalized confirmed COVID-19 patients, with various demographic and comorbidity profiles. One hundred and sixty-two (62.31%) of them were not vaccinated, and 98 (37.69%) were vaccinated. Out of the vaccinated group only 15 patients (5.77%) received two doses of the vaccine.

The effect of single vs. two vaccine shots

In the current study, we found that even a single vaccine dose is effective in preventing severe COVID-19 infection-related consequences, the adjusted odds ratio of death in the vaccinated group was 0.39 (CI is 0.15 to 0.93). The percentages of survival after single and two doses were 88% (73 out of 83 patients), and 80% (12 out of 15 patients), respectively. Of note, the majority of those who received two doses were diabetic, elderly, and renal transplant groups.

A case-control study conducted in England analyzed 156,930 adults aged 70 years and older showed that the BNT162b2 vaccine effects after the first dose, reached 70% (95%CI 59% to 78%), after the second dose vaccination effectiveness of 89% (85% to 93%). For the Oxford-AstraZeneca vaccine, effects were seen from 14 to 20 days after vaccination, reaching effectiveness of 60% (41% to 73%) from 28 to 34 days increasing to 73% (27% to 90%) from day 35 onwards [17].

Another case-control study done in Qatar showed that the effect was negligible in the first two weeks after the first dose. It increased to 36.8% (95% CI, 33.2 to 40.2) in the third week after the first dose and reached its peak at 77.5% (95% CI, 76.4 to 78.6) after the second dose [18].

Effect of COVID-19 vaccine on old patients

The risk of severe COVID-19 illness increases with age; this is why the CDC recommends that adults older than 65 years have the priority for receiving COVID-19 vaccines.

In our analysis, out of 260, only 51 (20%) patients aged 65 years and older were admitted with COVID-19 infection. 64.7% (n=33) of them were unvaccinated, death reported to be 54.55% (n=18) in this group. The vaccinated group contained 18 patients with a mortality of 33.33% (n=6). This analysis found that the COVID-19 vaccines are effective against hospital admission of COVID-19 in older patients and against death. This result is consistent with many studies; one project assessed BNT162b2 vaccine effectiveness in elderly patients, recruited 7,280 COVID-19 patients (5,451 [75%] were unvaccinated, 394 [5%] were fully vaccinated, and 867 [12%] were partially vaccinated). The vaccine effectiveness in patients aged 65-74 years was estimated to be 96% (95% CI = 94%-98%) and 84% (95% CI = 76%-89%), respectively. The efficacy in those aged ≥75 years was noted to be lower; 91% (95% CI = 87%-94%) and 66% (95% CI = 48%-77%) following full and partial vaccination, respectively [19]. This proves the high efficacy of full as well as partial vaccination in preventing hospitalization in the old population.

Effect of COVID-19 vaccine on patients with chronic conditions

In our project, 67.34% (n=66) vaccinated patients with COVID-19 infection were found to have one or more comorbidities including diabetes mellitus, hypertension, chronic obstructive lung disease, renal impairment, and cardiac diseases. We noticed that the efficacy of COVID-19 vaccines is high; mortality was estimated by 16.67%, 15%, 18.18%, 44.44%, and 15.38%, respectively, in comparison with the unvaccinated patients who had higher mortality rates of 35.71%, 33.33%, 62.5%, 63.64%, and 43.75%, respectively. The great efficacy of the vaccination was seen not only on healthy patients but also in individuals with multiple comorbidities. In one trial conducted in patients with underlying chronic medical diseases to assess the effectiveness of the BNT162B2 vaccine, a similar efficacy of the vaccine in the medically free group was observed (94.7% [95% CI, 85.9 to 98.6] and 95.3% [95% CI, 87.7 to 98.8]) in the group with known chronic diseases. Although the data are still limited regarding the efficacy of the COVID-19 vaccine on patients with underlying medical conditions, all the available studies show that the vaccination is beneficial and helps in lowering the infection severity and associated mortality in patients with chronic medical conditions [20,21].

Effect of COVID-19 vaccine on patients with cancer

Very few data are available regarding vaccine safety and efficacy in patients with immunocompromising conditions, such as cancer, or who take immunosuppressive therapies. The available data on the BNT162b2 vaccine in cancer patients suggest that vaccination is safe, but efficacy may be compromised, especially in those with hematologic malignancies. A prospective observational study included 151 patients with cancer (95 with a solid tumor and 56 with a hematologic malignancy), and 54 healthy controls without cancer, assessing the safety and immunogenicity of the BNT162b2 in patients with cancer and healthy controls. In the cancer group, one dose of the BNT162b2 vaccine yielded poor efficacy. However, a remarkable increase was observed in immunogenicity in patients with solid cancer within two weeks of a vaccine boost at day 21 after the first dose [22]. Several other studies showed similar results of suboptimal vaccine response in patients with hematologic malignancies [23-25].

In our study, the sample was small, hence, we could not accurately assess the mortality. We recruited a total of seven patients with cancer on immunosuppression therapy. Only two of these were vaccinated and both were discharged home in good condition, while two unvaccinated patients died.

Effect of COVID-19 vaccine on solid organ transplanted patients

Our study included only seven post-organ transplant patients (six kidneys and one liver) who were on active immunosuppressive therapy, six out of which were vaccinated (two of them received two doses). Unfortunately, three of the vaccinated group died, with an estimated mortality odds ratio of 10.166 (CI 1.151 to 89.755). This implies the efficacy of the vaccine on the transplantation population might be poor, whether after a single or two doses. The low vaccine efficacy among this group of patients is highly correlated to the minimal immune response following the vaccine, and this could be explained by the aggressive immunosuppressive therapies, low eGFR, and being elderly. One study done in France analyzed 101 post-solid organ transplant patients showed that the prevalence of the anti-COVID-19 antibodies that should be acquired after the vaccination was only 4% (95% CI, 1 to 10; four of 101 patients) after the first dose of vaccine, and 40% (95% CI, 31 to 51; 40 of 99 patients) following the second dose. However, four weeks after the third dose it reached up to 68% (95% CI, 58 to 77; 67 of 99 patients), indicating a significant improvement in immunogenicity following the booster third dose [26,27].

Effect of COVID-19 vaccine on obesity

Obesity is one of the comorbidities leading to the development of other chronic diseases, and hence, was found to be a recognized risk for severe COVID-19 infection. One theory attributed this to the presence of the ACE2 receptors in many adipose tissues, which serve as the access point for the virus to enter human cells leading to a higher viral load and a long time of viral clearance. Moreover, it increases inflammatory cytokines which contribute to more severe infection [28,29,30]. In our study, 25 vaccinated patients labeled as obese with body-mass index (BMI) of ≥ 30 kg/m^2^, 24% (n=6) died. Of note, most of the dead patients were older than 65 years, were post-renal transplantation, or had other comorbidities, such as renal impairment and diabetes mellitus. A retrospective cohort study was conducted in France on 124 obese patients showed a correlation between the need for invasive mechanical ventilation due to severe COVID-19 infection and obesity with a BMI of 30 kg/m^2^ and higher [31]. To date, we are lacking data regarding the effectiveness of COVID-19 vaccination in an obese population. In a multinational, placebo-controlled trial, the BNT162b2 was 95% effective in preventing COVID-19 (95% CI; 90.3 to 97.6). Similar vaccine efficacy was observed across subgroups, including patients with BMI ≥ 30.0 kg/m^2^ [12]. Thus, this group of patients should be encouraged to get vaccinated against COVID-19 infection.

Effect of COVID-19 vaccinations on high-flow oxygen need, ICU admission, and intubation

Whether the use of high-flow oxygen in patients with COVID-19 associated with acute respiratory failure improves clinically relevant outcomes remains unknown [32]. Out of 260 COVID-19 positive patients, only 104 patients required either invasive or non-invasive mechanical ventilation or 10 L or more of supplemental oxygen. However, the use of high-flow oxygen was shown to be more necessitated in the unvaccinated group than in the vaccinated group; 69 unvaccinated patients (42.59%) required high-flow oxygen, compared to only 35 vaccinated patients (35.71%). The vaccinated group showed less demand for high-flow oxygen than did the unvaccinated group. Critically ill COVID-19 patients in the emergency department or on the general ward were transferred to ICU for critical close monitoring or intervention. In our sample, 52 COVID-19 patients were transferred to the ICU, revealing a discrepancy between the unvaccinated and the vaccinated groups, where almost 37 unvaccinated patients (22.94%) required ICU admission, and 15 vaccinated patients (15.31%) were admitted to the ICU. Similarly, among the ICU admissions, 46 patients were intubated; 14 vaccinated patients (14.29%) and 32 unvaccinated patients (19.75%) required intubation.

Therefore, the vaccinated group reported less need for intubation, as well as fewer ICU admissions when compared to the unvaccinated group who, overall, needed more critical monitoring and intervention.

A recent study by Thompson et al. reported that the effectiveness of the full dose of mRNA-based COVID-19 vaccination in adults 50 years old or older was 89% (≥14 days after completing the full dose) against COVID-19 disease necessitating hospitalization. Among 7,283 COVID-19 positive patients, the effectiveness against COVID-19 leading ICU admission was reported to be 90% (95% CI, 86 to 93) [33].

Effect of COVID-19 vaccinations on mortality

Considering the transmissible nature of COVID-19, the epidemiological data report fluctuation, especially in the mortality rate. In Saudi Arabia, the incidence of COVID-19 and the mortality rate have been gradually declining, reflecting the implementation of successful preventive measures, and effective healthcare practices and treatment protocols.

As for our study analysis, reported deaths were 48 COVID-19 positive patients, of whom 35 patients (21.6%) were unvaccinated, while 13 patients (13.27%) were vaccinated either by one dose or the full dose.

A study by Bernal et al. showed evidence of the effectiveness of the Pfizer-BioNTech BNT162b2 and Oxford-Astra-Zeneca ChAdOx1-S vaccinations against symptomatic COVID-19 disease, hospitalization, and death in the older population. The vaccinated group had a 44% decreased risk of hospitalization and a 51% lower risk of death compared to the unvaccinated group [17].

There are multiple limitations to our study. First, it was conducted only at one health center for a two-month duration, hence the small sample size. Furthermore, our sample did not include a good number of transplant and cancer patients.

## Conclusions

Having only 98 vaccinated patients requiring hospital admission, compared to the huge number of vaccinated people in the region, implies the positive impact of the COVID-19 vaccine in preventing the infection as well as decreasing the severity of symptoms, and therefore the need for hospitalization. In our study, we demonstrated a statistically significant reduction in fatality among the vaccinated group, where even a single dose was effective in preventing major sequels.

We determined the vaccine effectiveness by having a mild hospital course, in terms of requiring less airway and oxygen support, endotracheal intubation, ICU care, and lower mortality rates. A reduced efficacy and poor response were observed in the elderly and those with chronic kidney disease and a history of renal transplant. Ultimately, we advise vaccination against COVID-19 infection for all eligible patients in keeping with the FDA recommendation.
